# Evolutionary Origin of the Carotenoid Cleavage Oxygenase Family in Plants and Expression of Pepper Genes in Response to Abiotic Stresses

**DOI:** 10.3389/fpls.2021.792832

**Published:** 2022-01-21

**Authors:** Yixiu Yao, Li Jia, Yuan Cheng, Meiying Ruan, Qingjing Ye, Rongqing Wang, Zhuping Yao, Guozhi Zhou, Jia Liu, Jiahong Yu, Peng Zhang, Yuhe Yin, Weiping Diao, Hongjian Wan

**Affiliations:** ^1^State Key Laboratory for Managing Biotic and Chemical Threats to the Quality and Safety of Agro-Products, Zhejiang Academy of Agricultural Sciences, Hangzhou, China; ^2^Institute of Vegetables, Zhejiang Academy of Agricultural Sciences, Hangzhou, China; ^3^Institute of Horticulture, Anhui Academy of Agricultural Sciences, Hefei, China; ^4^Wulanchabu Academy of Agricultural and Forestry Sciences, Wulanchabu, China; ^5^Jiangsu Key Laboratory for Horticultural Crop Genetic Improvement, Institute of Vegetable Crops, Jiangsu Academy of Agricultural Sciences, Nanjing, China; ^6^Australia-China Research Centre for Crop Improvement, Zhejiang Academy of Agricultural Sciences, Hangzhou, China

**Keywords:** pepper, phylogenetic analysis, gene expression, abiotic stress, NCED

## Abstract

Plant carotenoid cleavage oxygenase (CCO) is an enzyme that catalyzes the synthesis of carotenoids and participates in many important physiological functions. The plant CCOs exist in two forms, namely carotenoid cleavage dioxygenase (CCD) and nine-*cis* epoxide carotenoid dioxygenase (NCED). Although studies have shown that this gene family has been identified in many species, such as *Arabidopsis*, grape, and tomato, the evolutionary origin of the CCO family and the expression pattern of pepper genes in response to H_2_O_2_ and other abiotic stresses are still unclear. In this study, we used the bioinformatics method to identify and analyze the members of the *CCO* gene family from pepper and other 13 plants from lower to higher plant species based on the whole genome sequence. A total of 158 *CCO* genes were identified in different plant species and further divided into two groups (e.g., groups I and II). The former was subdivided into CCD7 and CCD8 and have independent evolutionary origins, respectively, while the latter was subdivided into CCD1, CCD4, CCD-like, and NCED, which may have come from a common ancestor. In addition, the results of RNA-seq showed that the expression patterns of pepper *CaCCO* genes were different in the tissues tested, and only few genes were expressed at high levels such as *CaCCD1a*, *CaCCD4a*, *CaNCED3*, and *CaCCD1b*. For hydrogen peroxide (H_2_O_2_) and other abiotic stresses, such as plant hormones, heat, cold, drought, and NaCl treatments, induction of about half of the *CaCCO* genes was observed. Moreover, the expression patterns of CaCCOs were further investigated under heat, cold, drought, and NaCl treatments using quantitative real-time PCR (qRT-PCR), and most members were responsive to these stresses, especially some CaCCOs with significant expression changes were identified, such as *CaCCD4c, CaCCD-like1, CaCCD8*, and *CaCCD1b*, suggesting the important roles of CaCCOs in abiotic stress responses. All these results will provide a valuable analytical basis for understanding the evolution and functions of the CCO family in plants.

## Introduction

As is known to all, due to the global climate change, plant resources and genetic diversity as well as the world food security has had a certain impact, so environmental stress has become the focus of attention of people (Raza et al., 2020). Plants are often subjected to various environmental stresses during their life, such as drought, high salt, high temperature, or invasion of pathogenic bacteria. These abiotic stresses or biotic stresses will have a certain impact on the growth and development of plants (Roychoudhury et al., 2013). Carotenoids are important lipid-soluble compounds, contain a large family over 700 types of structures (Britton et al., 2004), and perform a series of important biotic and abiotic stress functions. Apocarotenoids, or carotenoid cleavage products, were produced by carotenoid cleavage oxygenases (CCOs) of specific cleavage of carotenoids. Plant CCOs are a class of dioxygenases that catalyze the cleavage of carotenoids and their conjugate double bonds in plants. The CCOs can be further divided into nine-*cis*-epoxide carotenoid dioxygenase (NCED) and carotenoid cleavage dioxygenase (CCDs), based on their substrate informing an epoxy structure (Tan et al., 2003; Auldridge et al., 2006).

The first *NCED* gene *vp14* was identified in maize (Schwartz et al., 1997; Tan et al., 1997). Since then, the function of *NCED* gene has been widely studied. The *NCED* genes in these species exist in plants in the form of gene family (Yang and Guo, 2007), and their expression sites and functions are also different (Lefebvre et al., 2010). It is reported that the *NCED* genes play a certain role in the growth and development of plants. For example, in *Arabidopsis*, the *NCED1* gene can respond to water stress (Blankenship and Sisler, 1993). Similarly, according to the phenotypic analysis of *NCED5*, *NCED6*, and *NCED9* mutants at specific time and tissue, the results show that the three genes work together to induce seed dormancy (Frey et al., 2012). Seo et al. (2009) showed that spatiotemporal expression of the *NCED* gene is particularly important for the regulation of abscisic acid (ABA) level, which affects the seed dormancy and germination (Nambara et al., 2010). Furthermore, the inhibition of far-red light on *Arabidopsis* seed germination was reported in part due to the photoreversible regulation of *NCED6* to maintain the ABA levels (Seo et al., 2006), and Toh et al. (2008) suggested that three different *NCED* genes, namely, *NCED2*, *NCED5*, and *NCED9*, inhibited germination by increasing the ABA levels at high temperatures. Finally, two (*PaNCED1* and *PaNCED3*) of three avocado *NCED* genes cloned were highly expressed at ripening (Chernys and Zeevaart, 2000).

In addition, studies have shown that the *NCED* gene not only involves in the growth and development of plants but also plays an important role in their stress tolerance (Gómez et al., 2002). It has been reported that the *NCED* gene can upregulate ABA biosynthesis (Chernys and Zeevaart, 2000; Rodrigo, 2006). Overexpression of the *NCED* gene can enhance the resistance of plants to abiotic stresses (Xian et al., 2014). For example, overexpression of the *AtNCED3* gene can increase the content of ABA and increase the water loss tolerance of *Arabidopsis thaliana*. Previous reports have shown that overexpression of the *LeNCED1* gene can improve the drought tolerance of tomato (Thompson et al., 2000). The heterologous overexpression of *MhNCED3* in *Malus crabapple* alleviated oxidative damage and enhanced the tolerance of *A. thaliana* to chlorine stress (Zhang et al., 2014, 2015). The real-time PCR (RT-PCR) analysis of *OsNCED3*, *OsNCED4*, and *OsNCED5* on the root of rice seedlings showed that the three *NCED* genes were induced by salt and ABA treatments (Welsch et al., 2008). More recently, Hwang et al. (2018) studied the sugar sensitivity and drought tolerance of the allograft expression of rice *OsNCED4* in *A. thaliana* and found that the allograft expression of *OsNCED4* increased ABA levels, changed plant size and leaf shape, and delayed seed germination, leading to oversensitivity to sugar after germination and enhanced tolerance to drought. There are few reports on the involvement of the *NCED* gene in disease resistance regulation, but some studies have found that *AtNCED5* was overexpressed in *A. thalli* mutants, the endogenous ABA level was increased by about 2 times, and the susceptibility of this mutant was significantly improved. After inoculation with *Pseudomonas syringa*, *AtNCED2*, *AtNCED3*, and *AtNCED5* were significantly induced, and ABA was also accumulated in large quantities (Fan et al., 2009).

Hydrogen peroxide (H_2_O_2_) and NO are important signals involved in plant growth and abiotic stress tolerance (Neill et al., 2002; Uchida et al., 2002). The exogenous ABA treatment can induce the production of H_2_O_2_ and NO, resulting in stomatal closure, and improve the expression and activity of antioxidant enzymes (Jiang and Zhang, 2003; Desikan et al., 2004; Zhou et al., 2005; Zhang et al., 2007). Especially, research reported that rice *OsNECD3* regulates plant growth and enhances abiotic stress tolerance. Huang et al. (2018) showed that under H_2_O_2_ stress, *OsNCED3* was expressed in different tissues, and the *OsNCED3* gene avoided oxidative damage by increasing ABA biosynthesis when the plant was subjected to oxidative stress. Other studies have pointed out that the expression of the *NCED* gene increased the ABA and the H_2_O_2_ contents in roots in the non-saline side increased. Exogenous H_2_O_2_ can reduce ABA content by downregulating the *NCED* gene, indicating that there is a feedback mechanism between ABA and H_2_O_2_ (Kong et al., 2016).

Compared with the *NCED* gene family, only few studies were reported on the *CCD* gene family. Researchers reported that each member of this gene family had formed a functional carotenoid, such as β-ionone and geranylacetone, which were vital for the flavor of vegetable and fruit plants (Simkin et al., 2004a,b; Sun et al., 2008; Ilg et al., 2009). The *CCD1* enzymes have cleavage activities on 5,6 (5’, 6’) double bonds position, while *CCD4* were also found to have a 9,10 (9’, 10’) cleavage activity (Huang et al., 2009; Campbell et al., 2010; Brandi et al., 2011). In addition, the researchers reported that the cooperation of *CCD7* and *CCD8* can regulate plant architectures and reproductive development (Snowden et al., 2005; Liu et al., 2013).

Presently, nine members of the *CCO* gene family have been identified in *Arabidopsis*: four of which were designed as CCDs (*CCD1*, *CCD4*, *CCD7*, and *CCD8*) and the remaining genes were proven to be NCEDs (*NCED2*, *NCED3*, *NCED5*, *NCED6*, and *NCED9*) (Schwartz et al., 1997). In recent years, with the completion of plant genome sequencing, this gene family has been identified in different plant species, such as tomato (Burbidge et al., 1999), avocado (Chernys and Zeevaart, 2000), and bean (Qin and Zeevaart, 2002). Although identification and the roles of many CCOs in plants have been completed, members of the *CCO* gene family in pepper “zunla-1” have not been reported. Recently, the whole genome of “zunla-1” in pepper was sequenced (Qin et al., 2014). In this study, we systematically surveyed the *CCO* gene family from 14 plant species, ranging from lower to higher plant species, and analyzed their evolutionary origins. In addition, the expression profiles of the members of the pepper *CCO* gene family in response to H_2_O_2_ and other biotic stresses were also further investigated. These results will provide the value information of understanding of evolutionary modes and theoretical basis for exploring the functional roles of *CCO* in pepper.

## Materials and Methods

### Data Retrieval of Carotenoid Cleavage Oxygenase Gene Families in Different Plant Species

To identify the *CaCCO* gene family members, the predicted pepper gene sequences were downloaded from the Pepper Genome Database^1^ ^,2^. The genome information of *Arabidopsis* was derived from the database^3^. In addition, twelve plant species were also selected for analyses, including *Volvox carteri*, *Chlamydomonas reinhardtii*, *Marchantia polymorpha, Physcomitrella patens*, *Selaginella moellendorffii*, *Oryza sativa*, *Zea mays*, *Solanum lycopersicum*, *Brachypodium distachyon*, *Setaria italic*, *Aquilegia coerulea*, and *Solanum tuberosum*. Genome sequences of these plant species were downloaded from JGI^4^.

### Identification and Chromosome Localization of the Carotenoid Cleavage Oxygenase Gene Family in Pepper

The *CCO* gene sequences were downloaded from *A. thaliana* genome database and used as a seed sequence to retrieve the pepper genome database to obtain the candidate genes. To confirm the members of the pepper *CCO* family, BlastP methods were performed using the conserved domain of *Arabidopsis* CCO protein as a query sequence in the local database. The pepper *CCO* genes with e-value less than 1e − 5 were used for further analysis. Then, the Hidden Markov Model (HMM) profile of the PF03055 (RPE65) conserved domain was downloaded from the Pfam protein family database^5^ with e-value < 1e − 5. Finally, all of the putative CaCCO sequences with incomplete domains were excluded by the HMM analysis. Genome sequences of other 12 plant species were used to construct the local database using Bioedit 7.0 software^6^.

The physicochemical properties of *CaCCO* genes were analyzed using the online ExPASy-ProtParam tool^7^, which included several amino acids, molecular weight (MW), and theoretical isoelectric point (p*I*) of deduced CaCCO proteins. According to the information in the pepper gene database, chromosome localization of the *CCO* genes was further performed using MapDrawV2.1 software.

### Phylogenetic Analysis of Carotenoid Cleavage Oxygenase Gene Family in Pepper and Other Plant Species

To explore the phylogenetic relationships of the pepper *CCO* genes, multiple sequence comparison was conducted using CCO amino acid sequences of pepper and other plant species using ClustalW program, and MEGA5.0 software^8^ was used to construct neighbor-joining (NJ) tree (Ilg et al., 2009).

### Spatiotemporal Expression Analysis of CaCCO Genes in Various Tissues of Pepper

To study the spatiotemporal expression patterns of the pepper *CCO* genes, data of reported RNA-seq were selected for analyses (see text footnote 2). Previously, a pepper variety (Line 6421) with good heat resistance, drought resistance, and disease resistance was selected as the preparation materials of various samples for RNA sequencing. The seeds were surface disinfected with 5% sodium hypochlorite solution for 15 min. After rinsing with water, the seeds were sown in 200-well vermiculite-filled seedling trays and placed in an environment of 25/18*^o^*C day/night temperature, 16/8 h light/dark cycle, 60--70% relative humidity, and 6,000 lux light intensity. The RNA-seq sequencing data of various tissue development stages of pepper, including seeds and placenta (ST1--ST2), seed (S3--S11), placenta (T3--T11), flower (F1--F9), petal (P10), ovary (O10), stamen (STA10), leaf (L1--L9), fruits (FST0--FST1), and peel (G1--G11), were used to make heat maps, and the heat map of tissue-specific expression patterns of the *CCO* genes was drawn using the MeV software^9^. All samples were collected in quadruplicate from each of the sampling points, and five seedlings were randomly picked and mixed as one biological replicate.

### Expression Analysis of CaCCO Genes Under Abiotic Stresses

Similarly, to determine the response of the *CaCCO* gene to abiotic stresses (heat, cold, drought, and NaCl treatments), 40-day-old seedlings were treated and RNA samples were extracted from leaves and roots. Salt stress was carried out by adding NaCl with a final concentration of 200 mM to the nutrient solution, and osmotic stress was carried out by adding mannitol with a final concentration of 400 mM. For high/low-temperature treatment, the seedlings at the fourth true leaf stage will be transferred to the 42*^o^*C (high temperature) or 10*^o^*C (low temperature) of the growth chamber, photoperiod, and relative humidity consistent with the untreated plants. The control was treated with nutrient solution only. The leaves and roots were selected for RNA-seq analysis at 0, 1, 6, and 24 h after treatment.

### Expression Analysis of CaCCO Genes in Response to Hormone Stresses

To study the expression of these genes under different hormone treatments, 30 μM ABA, 2 mM salicylic acid (SA), and 10 μM methyl jasmonic acid (MeJA) were used to treat the seedling for 40 days. These three different hormones (ABA, MeJA, and SA) were selected and added to the nutrient solution. The leaves and roots were selected for RNA-seq analysis (see text footnote 2) at 0, 1, 6, and 24 h after treatment. An isometric histogram was drawn using OriginPro7.5^10^.

### Expression Analysis of CaCCO Genes in Response to H_2_O_2_ Stress

The oxidative stress was also applied by adding H_2_O_2_ into the nutrient solution to a final concentration of 30 mM. Then, the leaves and roots were selected for RNA-seq analysis (see text footnote 2) at 0, 1, 6, and 24 h after treatment. The heat map of expression patterns of *CCO* genes in response to H_2_O_2_ treatment was drawn using the MeV software.

### RNA Isolation and Quantitative Real-Time PCR Analysis

Total RNA was extracted from the pepper leaves using E.Z.N.A.^®^ Plant RNA Kit (OMEGA, United States). The first strand cDNA was synthesized using FastKing RT Kit (with gDNase) (TIANGEN, China) according to the instructions of the manufacturer. Gene-specific primers were designed using the Genscript online tool^11^, and the detailed information is listed in Supplementary Table 2. The pepper *GAPDH* gene was utilized as an internal control for normalizing the expression levels. The use of primers was diluted according to the synthetic instructions. The reaction system followed the instructions of SYBR Green Master Mix reagent of Vazyme using CFX96 Real Time System (Bio-Rad, United States) with AceQ^®^ qPCR SYBR^®^ Green Master Mix with 20 μl reaction mixture of volume. The reaction volume consists of 10 μl SYBR^®^ Green Master Mix, 0.4 μl of each primer (10 μM), 1 μl of the cDNA template, and 7.8 μl of RNase free H_2_O. Thermal cycling parameters for the amplification were as follows: 95°C, 10 min, followed by 40 cycles at 95°C, 15 s, and 55°C, 1 min. Three technical repeats were performed for each gene, and the data were analyzed according to the 2^–ΔΔCt^ method.

## Results

### Identification of the CaCCO Gene Family in Pepper and Other Plant Species

To identify the members of the *CCO* gene family at the genome-wide levels, the whole genome sequence of pepper was searched using HMM and BLAST. The results showed that a total of 158 candidate *CCO* genes were identified in various plant species, each with a distinct number of variants. In addition, fourteen CCO DNA binding domains containing proteins were encoded in the pepper genome. The detailed information of these proteins is shown in Table 1, including gene name, genome location, coding sequence length, amino acid sequence length, MW, and isoelectric point. The predicted size of the CaCCO proteins was from 321 to 744 AA (for *CaCCD4b* and *CaCCD7*, respectively; Table 1). The MW of these genes ranged from 36.33 (*CaCCD4b*) to 83.79 kDa (*CaCCD7*), while their estimated p*I* values ranged from 5.16 (*CaCCD4b*) to 8.25 (CaNCED1). Most CaCCO proteins were acidic (p*I*-values 7) according to the p*I* values.

**TABLE 1:** Information for the *CaCCO* gene family in pepper.

	Gene name	Gene ID	Protein length (AA)	Molecular weight (kD)	Isoelectric point
CaCCD	CaCCD1a	Capana01g002623	581	65.81	6.49
	CaCCD1b	Capana01g002624	565	63.14	5.86
	CaCCD4a	Capana01g000948	603	66.05	6.34
	CaCCD4	Capana01g000946/Capana01g000947	321	36.33	5.16
	CaCCD4c	Capana01g004038	568	63.13	7.85
	CaCCD7	Capana01g003456	744	83.79	6.67
	CaCCD8	Capana08g000570	501	56.15	6.04
	CaCCD-like1	Capana08g000589	567	64.06	6.18
	CaCCD-like2	Capana11g000021	565	64.72	5.56
	CaCCD-like3	Capana11g000005	442	49.35	5.49
CaNCED	CaNCED1	Capana05g002276	579	64.67	8.25
	CaNCED2	Capana01g003704	596	66.49	6.64
	CaNCED3	Capana00g003114	506	56.47	5.6
	CaNCED4	Capana03g002722/Capana03g002723	353	40.04	6.16

### Evolutionary Origin of Carotenoid Cleavage Oxygenase Gene Family in Plant Species

**FIGURE 1:**
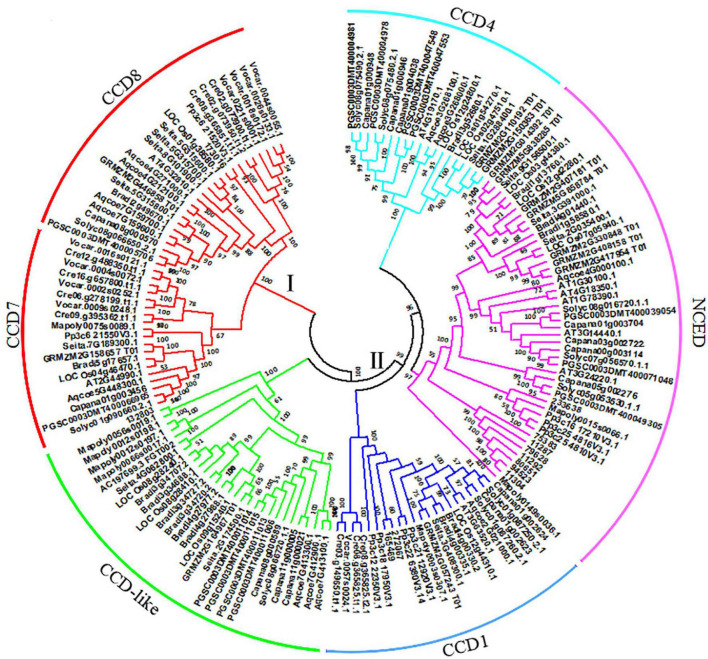
Evolutionary origin of carotenoid cleavage oxygenase (CCO) proteins in plant species. The unrooted tree was constructed using the neighbor-joining (NJ) method in MEGA5.0 software. The parameters were 1,000 bootstraps. The CCO proteins were classified into two groups: I and II. The former was subdivided into CCD7 and CCD8, while the latter was subdivided into CCD1, CCD-like, CCD4, and NCED.

To investigate the evolutionary origin, a total of 158 CCOs (CCDs and NCEDs) from 14 plant species were identified (Supplementary Table 1). A phylogenetic tree was constructed based on the amino acid sequences of these genes using the NJ method in MEGA 5.0 software (Tamura et al., 2011). As shown in Figure 1, all of the CCOs in different plants ranging from green algae to higher plant species were divided into two groups (groups I and II), and the former was further separated into two subgroups (*CCD7* and *CCD8*). Both of these two subgroups contained the members from the lower to higher plant species, suggesting that they have independent evolutionary origins, respectively. Inversely, the latter was subdivided into *CCD1*, *CCD4*, *CCD-like*, and *NCED*. The *CCD1* subgroup was composed of members from green algae to higher plant species, both *NCED* and *CCD-like* subgroups contained *CCO* genes from moss to higher plant species, and *CCD4* was composed of *CCO* genes from angiosperm. This showed that group II may have come from a common ancestor, and *NCED*, *CCD-like*, and *CCD4* were produced from multiple duplication events in land plant species.

### Chromosome Distribution of the CaCCO Gene Family Members

**FIGURE 2:**
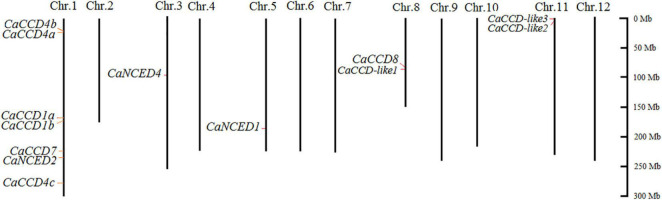
Chromosomal mapping of the *CCO* genes in *Capsicum annuum*. The chromosome number is shown at the top of each bar chart, while the size of the chromosome is shown in its relative length. The scale on the right is in MB, and the short line represents the approximate positions of the *CaCCO* genes on the respective chromosome.

In this study, the chromosome distribution of the pepper *CaCCO* gene was analyzed based on the genome sequence of the pepper. Except for *CaNCED3*, the remaining thirteen genes in the fourteen CaCCO members were irregularly distributed on five of the twelve pepper chromosomes, according to the findings (Figure 2). Among them, most of the genes are located on the first chromosome (Chr1), and there are seven genes, namely, *CaCCD1a*, *CaNCED2*, *CaCCD4a*, *CaCCD1b*, *CaCCD4c*, *CaCCD7*, and *CaCCD4b*. The third and fifth chromosomes had only one member (*CaNCED4* and *CaNCED1*, respectively). Furthermore, two genes are found to be present on each of the two chromosomes (Chr8 and 11). None of the *CaCCO* genes was mapped on Chr2, 4, 6, 7, 9, 10, and 12. Subsequently, we further analyzed the tandem and segmental duplications of the *CaCCO* genes in pepper. We found that both of the tandem duplication and segmental duplication events were not found in the *CaCCO* gene family.

### Expression Analysis of CaCCO Genes in Different Tissues

**FIGURE 3:**
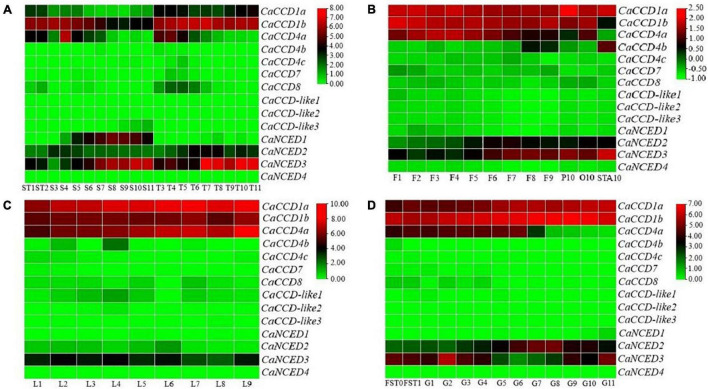
Expression patterns of CaCCOs in different tissues of pepper. Key: red, strong expression; black, weak expression; green, no expression. The expression data were hierarchically clustered based on MeV software. **(A)** Seeds and placenta, **(B)** flowers, **(C)** leaf, and **(D)** fruit and peel.

It is well known that RNA-seq data have a wide range of applications and can be used to conveniently analyze the differential expression of genes in higher plant species (del Río et al., 2006). In this study, the expression patterns of fourteen *CaCCO* genes were analyzed using RNA-seq data from seeds, leaves, flowers, and fruits. The results revealed that most of the *CaCCO* genes in these organs were completely unexpressed in all of the examined tissues (Figure 3). In different developmental stages of leaves from L1 to L9, expression levels of 10 of 14 *CaCCO* genes were not detected, including *CaNCED1*, *CaNCED2*, *CaCCD4c*, *CaCCD7*, *CaCCD*-*like1*, *CaCCD8*, *CaCCD*-*like2*, *CaCCD*-*like3*, *CaNCED4*, and *CaCCD4b*. Three genes (*CaCCD1a*, *CaCCD4a*, and *CaCCD1b*) were expressed constitutively in all of the stages that were analyzed. The expression of *CaNCED3* is weak and gradually decreases along with the development.

The expression patterns of all *CaCCO* genes in the flower organs were explored, including twelve different tested stages (F1–F9), petals (P10), ovary (O10), and stamens (STA10). As shown in Figure 3, among 14 *CaCCO* genes, three members (*CaCCD1a*, *CaNCED3*, and *CaCCD1b*) were expressed in all twelve tested stages. The expression levels of *CaNCED2* and *CaNCED3* increased gradually in different developmental stages, but those of *CaCCD4a* and *CaCCD1b* decreased gradually in different stages, and *CaCCD4a* was not expressed in stamens. *CaCCD1a* has the highest expression in all periods, while *CaCCD4b* only has the expression in F8, F9, and STA10, and the expression in STA10 is the highest.

In pepper fruit, three tissues (placenta, seeds, and pericarp) were selected for analyzing the expression patterns of the *CaCCO* genes. The developmental stages in each of the tissues (placenta: T3–T11, seed: S3–S11, pericarp: G1–G11, fruits at days 3 and 7: FST0, FST1, and seeds and placenta at days 10 and 15: ST1, ST2) were included. As shown in Figure 3, half members of the *CaCCO* gene family have relatively high expression levels, and the mean of the log-signal values of each gene is within the range of 10 to 20. On the contrary, nine genes show relatively low expression levels with the mean of the log-signal values of each gene ranging from 0 to 1.

In the placenta and seed, the results showed that most of the *CaCCO* genes were expressed in different developmental stages. Some genes such as *CaCCD1a*, *CaNCED2*, *CaCCD4a*, *CaNCED3*, and *CaCCD1b* were highly expressed in the seeds and placenta of capsicum, indicating that these genes may play an important role in the development of capsicum plants. The gene *CaCCD1a* was highly expressed in T3–T11, and *CaNCED1* was in S5–S11. The *CaNCED3* and *CaCCD1b* were highly expressed in ST1–T11. The transcript levels of *CaCCD1b* were highly expressed in S3. In pericarp, there were four members with high expression, namely, *CaCCD1a*, *CaCCD4a*, *CaNCED3*, and *CaCCD1b*. Among them, *CaCCD1a* and *CaCCD1b* have the highest expression in each stage of fruit development; *CaCCD4a* has a higher expression in the early stage of fruit development and a lower expression in the later stage, but not in the G11 stage. *CaCCD4c*, *CaCCD7*, *CaCCD*-*like1*, *CaCCD8*, *CaCCD*-*like2*, *CaCCD*-*like3*, *CaCCD4b*, and *CaNCED1* gene has a similar expression in different period of time, such as FST0–G11. The *CaCCO* gene has a lower expression. These results indicate that half of *CaCCO* genes are involved in plant growth and development.

### Expression of CaCCOs in Response to Hormone Treatments

Previous studies have shown that hormones, including cytokinins (CKs), gibberellins (GAs), salicylic acid (SA), jasmonic acid (MeJA), ethylene (ET), ABA, and brassinosteroids (BRs), play a key role in regulating plant growth and development, adapting to environmental stress and complex signal networks (Vallabhaneni et al., 2010). To further analyze the expression patterns of *CaCCO* genes under stress-related stimulation, the expression profiles of leaves and roots of *CaCCO* genes under ABA, MeJA, and SA-hormone treatments were analyzed.

**FIGURE 4:**
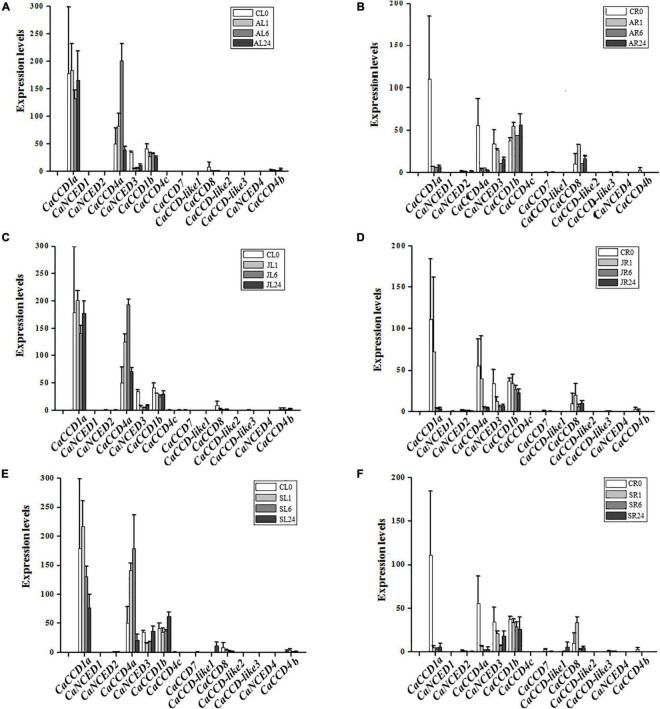
Expression of *CaCCO* genes under different hormone treatments. **(A)** Leaves under abscisic acid (ABA) treatment, **(B)** roots under ABA treatment, **(C)** leaves under jasmonic acid (MeJA) treatment, **(D)** roots under MeJA treatment, **(E)** leaves under salicylic acid (SA) treatment, and **(F)** roots under SA treatment.

As illustrated in Figure 4, we found that fourteen *CaCCO* genes have different expression patterns in roots and leaves under three different hormone treatments. Among them, five *CaCCO* genes had high expression levels, while the remaining nine genes had low or even no expression levels. The five genes were induced significantly by ABA, MeJA, and SA treatments. Among the five genes, the root expressions of *CaCCD1a* and *CaCCD4a* significantly decreased after treatment with ABA, MeJA, and SA for 1, 6, and 24 h, while the expression levels in leaves showed a trend of first increasing and then decreasing. Among them, the expression levels of *CaCCD4a* under treatment with different hormones were the highest at 6 h for ABA, MeJA, and SA, while the expressions of *CaCCD1a* and *CaNCED3* were in reverse manner. Expression levels were decreased at 6 h for ABA, MeJA, and SA.

### Expression of CaCCOs in Response to Cold, Heat, Drought, and NaCl Treatments

Abiotic stresses (heat, cold, drought, and salt) affected the plant growth and development and were the major factors limiting crop production (Simkin et al., 2004a; Ohmiya et al., 2006; Sun et al., 2008; Adami et al., 2013; Liu et al., 2013; Bai et al., 2017). To illustrate the roles of *CaCCO* gene family members in abiotic stresses, the RNA-seq data of pepper under four treatments (heat, cold, drought, and NaCl) have been selected. Interestingly, similar expression patterns of most of the *CaCCO* genes were observed in pepper, whereas some differences in expression patterns can be carefully shown. The results showed that at least four genes were significantly induced under different stresses, and these genes have similar expression patterns, such as *CaCCD1a*, *CaCCD4a*, *CaNCED3*, *CaCCD1b*, and *CaCCD8*. The remaining *CaCCO* genes are not regulated under low temperature, high temperature, drought, and salt stresses.

**FIGURE 5:**
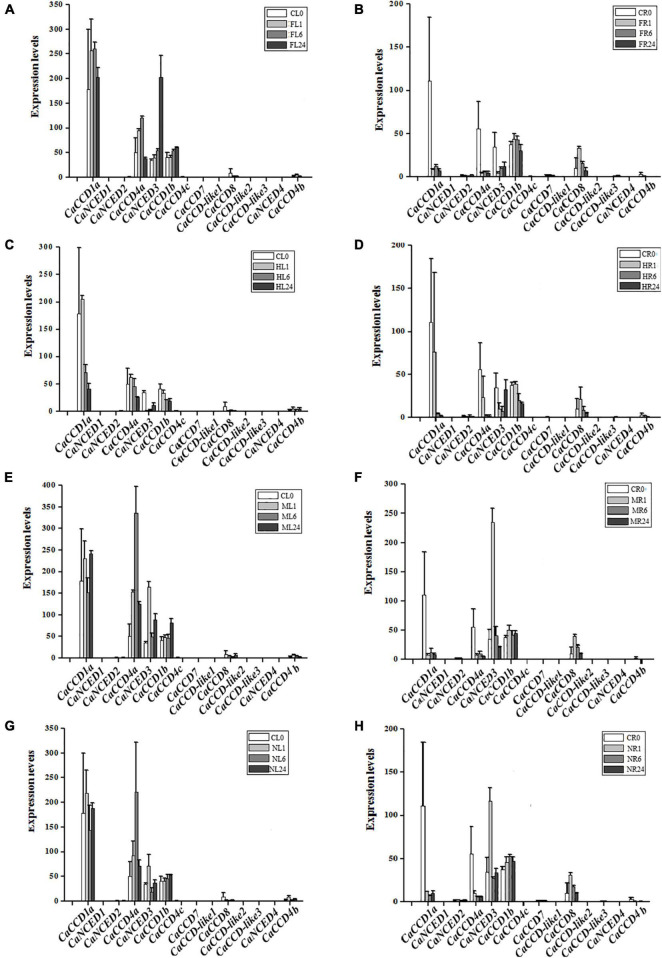
Expression patterns of *CaCCO* genes under low/high temperature, drought, and salt stresses. **(A)** Leaves under cold stress, **(B)** roots under cold stress, **(C)** leaves under heat stress, **(D)** roots under heat stress, **(E)** leaves under drought stress, **(F)** roots under drought stress, **(G)** leaves under NaCl stress, and **(H)** roots under NaCl stress.

For cold stress (Figure 5), *CaCCD1a* and *CaCCD4a* share a similar expression pattern, and the upregulated expression was followed by a downregulated expression in FL1–FL24. However, in roots, the expression of these two genes was downregulated after cold treatment. The expressions of *CaNCED3* and *CaCCD1b* were upregulated in leaves under low-temperature treatment, especially the expression level of *CaNCED3* was increased about 200 times after 24 h of low-temperature treatment. Under heat treatment conditions, the *CaCCD1a* and *CaCCD4a* slightly upregulated the stage of CL0 and HL1, then the *CaCCD1a* and *CaCCD4a* gene was downregulated in all the stages analyzed. However, in roots, the expression of these two genes was significantly decreased after high-temperature treatment.

Under drought stress (Figure 5), the expression of *CaCCD4a* in leaves was upregulated first and then downregulated, and the highest expression level was reached after 4 h of drought treatment. However, the expression of this gene was significantly downregulated in roots. The expression of *CaNCED3* was the highest in leaves and roots at 1 h after drought treatment and then decreased. Of the fourteen genes, about half responded to salt stress, suggesting that these genes were involved in coping with environmental changes. In leaves, a total of 6 genes were upregulated, among which the most significant ones were *CaCCD1a* and *CaCCD4a*, followed by *CaNCED3* and *CaNCCD1b*, and the expressions of *CaCCD8* and *CaCCD4b* were the lowest. The expression pattern of the *CaCCO* gene family in roots under salt stress was the same as that in roots under drought treatment, the expressions of *CaCCD1a* and *CaCCD4a* were significantly downregulated, and the expressions of *CaNCED3*, *CaCCD1b*, and *CaCCD8* were upregulated first and then downregulated.

### Expression of CaCCOs in Response to H_2_O_2_ Treatments

**FIGURE 6:**
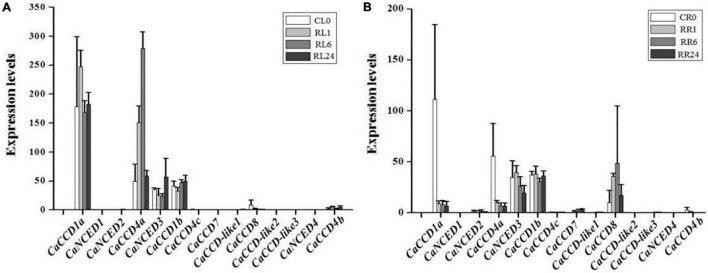
Expression of *CaCCO* genes under hydrogen peroxide (H_2_O_2_) treatment. **(A)** Leaves under H_2_O_2_ stress and **(B)** roots under H_2_O_2_ stress.

Under the H_2_O_2_ treatment condition (Figure 6), expressions of 6 *CaCCO* genes were induced in pepper leaf, including *CaCCD1a*, *CaCCD4a*, *CaNCED3*, *CaCCD1b*, *CaCCD8*, and *CaCCD4b*. Among them, the expression levels of *CaCCD1a* and *CaCCD4a* were first increased and then decreased. The expression level of *CaCCD1a* was the highest at 1 h of H_2_O_2_ treatment, while *CaCCD4a* reached the maximum at 6 h of H_2_O_2_ treatment. In addition, the trend of *CaNCED3* and *CaCCD1b* treated with H_2_O_2_ was opposite to the former. The expression levels reached a peak at 24 h after treatment. In root, expressions of 8 *CaCCO* genes were induced, namely *CaCCD1a*, *CaNCED2*, *CaCCD4a*, *CaNCED3*, *CaCCD1b*, *CaCCD7*, *CaCCD8*, and *CaCCD4b*. Among them, the expression levels of *CaCCD1a*, *CaCCD4a*, and *CaNCED3* decreased gradually, while *CaCCD1b* and *CaCCD8* increased first and then decreased. The expression levels of *CaCCD1b* reached the highest at 1 h and *CaCCD8* reached the peak at 6 h.

### Quantitative RT-PCR Analysis of Members of *CaCCO* Gene Family in Response to Abiotic Stresses

**FIGURE 7:**
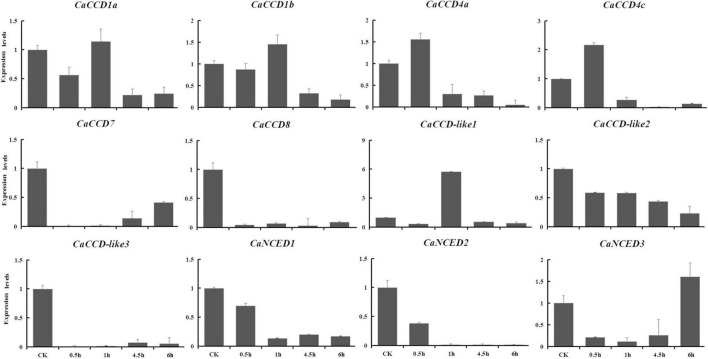
Expression patterns of *CaCCO* genes in response to heat stress at different time points.

**FIGURE 8:**
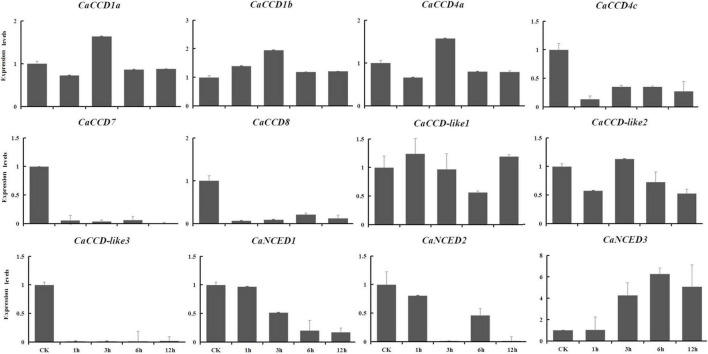
Expression patterns of *CaCCO* genes in response to cold stress at different time points.

The expression levels of the CaCCOs were investigated under heat, cold, drought, and NaCl treatments using qRT-PCR to understand their possible roles in responses to abiotic stresses (Table 2). Under heat stress, the expression levels of half of the genes were significantly downregulated with large-fold changes at the 0.5, 1, 4.5, and 6 h, respectively, while *CaCCD4a*, *CaCCD4c*, and *CaCCD*-*like1* were first upregulated and then downregulated at three of the four time points (Figure 7). Notably, some *CaCCDs* exhibited significant differences across the four time points. *CaCCD4a* and *CaCCD4c* reached the maximum value at 0.5 h. Three members (*CaCCD1a*, *CaCCD1b*, and *CaCCD*-*like1*) significantly elevated at 1 h. *CaNCED3* expression was significantly downregulated at 0.5 h, but significantly upregulated at 6 h after heat treatment. Under cold stress, we found that expression levels of *CaCCD4c*, *CaCCD7*, *CaCCD8*, *CaCCD*-*like3*, *CaNCED1*, and *CaNCED2* were significantly downregulated at all of the four time points (Figure 8). In addition, the expression levels of four genes, namely, *CaCCD1a*, *CaCCD1b*, *CaCCD4a*, and *CaNCED3*, showed a trend of upregulation and then downregulation, respectively. Notably, *CaCCD1a*, *CaCCD1b*, *CaCCD4a*, and *CaCCD*-*like2* all peaked at 3 h after cold treatment.

**TABLE 2:** Sequences of the primers used in quantitative real-time PCR (qRT-PCR) analysis in this study.

Gene name	Forward primers	Reverse primers
GAPDH	ATGATGATGTGAAAGCAGCG	TTTCAACTGGTGGCTGCTAC
CaCCD1a	GCGTCATGCAAGATCCAGTT	AAAGCGAGCCTTCTTTGTGG
CaCCD1b	TCGTCCGAAGGAAATGGTGA	CCAAGCGTTGGCATTATGGA
CaCCD4a	TCTTCCACGTGGTCCTTACC	AGGCAGAGAGACCGTTGAAA
CaCCD4c	TGGACCTAATTCGCCCAAGA	AGGGTTCCCAATAGCTGCAT
CaCCD7	GGATATGTTGCTCCCTCGGA	TGCTGTCATTGACCCAGGAA
CaCCD8	GTGTAAAGCCAGTGGCAACA	CGGAGATTGTCAAGGCGAAG
CaCCD-like1	GTGTTGAGGTTTGGCGTGAT	GCTTTCTTTGGGTGGCTTGT
CaCCD-like2	AAGATGAGGTGGTGGTGAGG	GAAGTTTGGCTAGCCCTCCA
CaCCD-like3	TGCGAGAATTGGAATAATGCCT	CTACTGCGATAGCCGGTCTT
CaNCED1	CTGCTTCTGCATGCATCTGT	CCGTTTGGTTGACTCACCTG
CaNCED2	GAAGAGCCAGAGACGGATGA	TCGTTTCCTCGACTCTCCAG
CaNCED3	TGGCATGGTTCATGCTGTTC	CCGAAGAGTCCACGAGCATA

**FIGURE 9:**
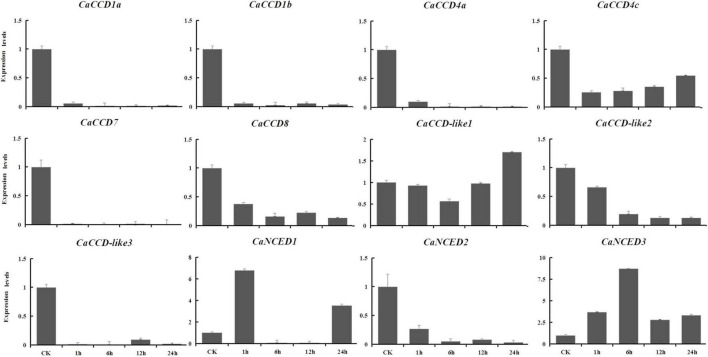
Expression patterns of *CaCCO* genes in response to drought stress at different time points.

**FIGURE 10:**
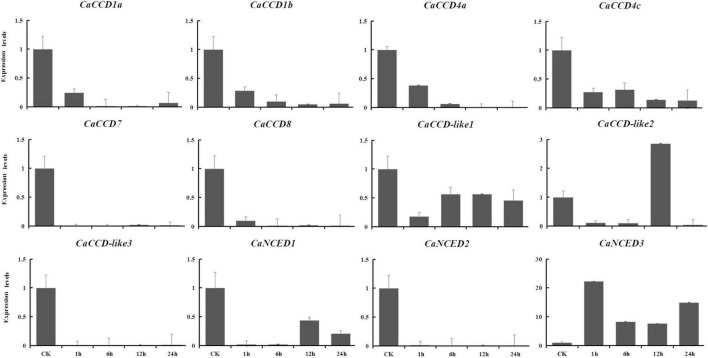
Expression patterns of *CaCCO* genes in response to salt stress at different time points.

To explore the expression patterns of CCO gene family in pepper in response to drought and NaCl stresses, based on the RNA-seq data, we showed the upregulated in leaves, selected to verify using qRT-PCR analysis at 1, 6, 12, and 24 h expression patterns after treatments. For drought stress, nine genes were downregulated (Figure 9), including *CaCCD1a*, *CaCCD1b*, *CaCCD4a*, *CaCCD4c*, *CaCCD7*, *CaCCD8*, *CaCCD*-*like2*, *CaCCD*-*like3*, and *CaNCED2*. In addition, only *CaNCED1* and *CaNCED3* showed upregulation before downregulation among the twelve genes. However, the difference between the two groups was that the expression level of the former reached its peak at 1 h under drought treatment, increased by 5.7 times compared with the control, while the latter reached its peak at 6 h and increased by 7.7 times compared with the control. In addition, under 400 mM NaCl treatment (Figure 10), our results found that the expression levels of most genes were downregulated, including *CaCCD1a*, *CaCCD1b*, *CaCCD4a*, *CaCCD4c*, *CaCCD7*, *CaCCD8*, *CaCCD*-*like1*, *CaCCD*-*like3*, *CaNCED1*, and *CaNCED2*. However, only *CaCCD*-*like2* and *CaNCED3* were upregulated and then downregulated. Notably, the expression of *CaCCD*-*like2* was the highest after 12 h of stress treatment, while the expression of *CaNCED3* was the highest after 1 h of stress treatment. These results showed that the *CaCCD* gene family has a certain function in plant response to abiotic stress, but its molecular mechanism may be different among different genes.

## Discussion

Plant *CCO* gene family, a class of specific enzymes, can catalyze conjugated double-bond system of carotenoids and their apocarotenoids to form smaller compounds (Ilg et al., 2009; Walter et al., 2010). These CCO proteins catalyze the cleavage of carotenoids and help to adjust the plant responses to stress (Espasandin et al., 2014). Therefore, it is very important to understand the evolutionary relationship and function of *CCO* genes in plant species. In this study, our results showed that plant *CCO* genes could be divided into two subfamilies (groups I and II). Group I was subdivided into *CCD7* and *CCD*, and group II was composed of *CCD1*, *CCD4*, *CCD-like*, and *NCED*. We found that orthology groups (*CCD7* and *CCD8*) appeared to be conserved only as single- or low-copy genes in all plants, whereas group II genes underwent several duplication events, resulting in multiple gene copies. These duplication events were due to whole-genome duplications in plants. Our results indicate that group II genes had strikingly different patterns of gene duplication from genes from *CCD7* and *CCD8*. The *CCD1* genes expanded during the histories of land plants (Figure 1). Unlike *CCO* genes from group II, other types of plant *CCO* genes (*CCD7* and *CCD8*) are single- or low-copy genes. The stably maintained low-copy numbers for these *CCO* genes suggest the functional conservation during the process of evolution in plants. These results also suggested that differential evolution of members of the *CCO* gene family with conservative and divergent patterns were observed.

The gene expression profiles often have some connection with gene function. To date, although the expression patterns of *CCO* genes have been determined in other plants, there are few detailed studies on the expression of *CCO* genes in pepper. To determine whether there were differences in the expression of *CaCCO* genes, the transcriptional levels of each member in pepper were analyzed spatially and temporally. In four different organs, about half of the *CaCCO* genes were not fully expressed at all stages of detection. In leaf, the *CaCCD1a*, *CaCCD4a*, *CaNCED3*, and *CaCCD1b* were expressed in all of the detected stages. Compared with the other three genes, the expression of *CaNCED3* was lower at all stages. We concluded that they are likely to act as a housekeeping gene of pepper cells under normal growth conditions. In flower, high expression levels of four genes (*CaCCD1a*, *CaCCD4a*, *CaNCED3*, and *CaCCD1b*) were observed in all the stages analyzed (F1–F9), indicating that these genes were involved in the growth and development of the flower. The *CaNCED2* and *CaNCED3* are transcribed at the late stage (F5–F9), while *CaCCD1a*, *CaCCD4a*, and *CaCCD1b* are transcribed in the whole development stage (F1–F9). These results suggest that these genes may play different functional roles at different stages of development.

We further analyzed the expression profiles of these *CaCCO* genes in the placenta, seed, and pericarp. In the placenta, all genes were expressed at different stages. Among them, the expressions of *CaCCD1a*, *CaNCED2*, *CaNCED3*, and *CaCCD1b* were the highest (T3–T11), and the expression of *CaNCED1* was the lowest in the whole placenta development period. The expression levels of *CaCCD4a* and *CaCCD8* were highest in the early placental stage (T1–T6) and decreased in the late placental stage (T7–T11). In seeds, *CaCCD4a* was expressed preferentially in the early stage (S1–S7), while *CaNCED1* was transcribed in the late stage (S5–S11). The expression level of *CaCCD1b* was the highest in each period, and the expression level of *CaNCED3* was the highest in all periods except S3–S4. The results indicated that these genes are related to seed development and maturation in pepper plants. The expression levels of two genes (*CaCCD1a* and *CaCCD1b*) in the pericarp were the highest (FST0–G11) during the whole development period. *CaCCD4a* and *CaNCED3* were expressed in the early stage (G1–G4), and *CaCCD4c* and *CaCCD1b* were expressed in the late stage (G5–G11). We concluded that these genes play important roles in the formation and maturity of pepper pericarp. In conclusion, these *CaCCO* genes may have specific functions in different organs and stages.

Under abiotic stress, the effects of heat and cold stress on plant growth induced the expression of multiple *CaCCO* genes in leaves. Under heat stress, *CaCCD1a*, *CaCCD4a*, and *CaCCD1b* genes were downregulated at different stages. Under cold stress, four *CaCCO* genes (*CaCCD1a*, *CaCCD4a*, *CaNCED3*, and *CaCCD1b*) were obviously induced, while *CaCCD8* and *CaCCD4b* were slightly induced. Drought stress and salt stress also induced this trend.

The NCED, a key rate-limiting enzyme in ABA biosynthesis, plays an important role in regulating plant growth and development and stress responses. Some studies have reported that ABA synthesis in plants can respond to oxidative stress, and oxidative reaction has the function of reactive oxygen species (ROS) clearance. ROS are biological molecules that are necessary for seed dormancy and germination. Among them, H_2_O_2_ can maintain cell homeostasis and participate in the regulation of photosynthesis, seed germination, and stomatal characteristics, and other physiological activities. O^2–^ and other ROS such as hydroxyl radical (OH), perhydroxy radical (HO^2^), alkoxy radicals (RO), H_2_O_2_, and singlet oxygen (^1^O_2_) are produced continuously as byproducts of various metabolic pathways that are localized in different cellular compartments including mitochondria, chloroplast, and peroxisomes (del Río et al., 2006; Navrot et al., 2007). Under stable conditions, ROS can be removed and maintained in equilibrium utilizing antioxidants or antioxidant enzymes (Gill and Tuteja, 2010). In the study, the results indicate that about half of the *CCO* genes can be induced by H_2_O_2_ stress in both roots and leaves, and their expression levels in leaves are upregulated first and downregulated later, which may be related to ABA synthesis and stress regulation. The specific mechanism of *CCO* genes needs to be confirmed by further experiments.

## Conclusion

The RNA-seq analysis of the *CaCCO* gene family provides a solid foundation for us to fully understand the functions of the *CCO* gene in pepper growth and development and abiotic stress. However, the functions of these genes in each genome are still unclear and need to be verified by further experiments.

## Data Availability Statement

The original contributions presented in the study are included in the article/Supplementary Material, further inquiries can be directed to the corresponding authors.

## Author Contributions

YXY, LJ, and HW conceived and designed the research. YC, MR, QY, RW, ZY, and GZ performed the experiments. JL, JY, PZ, and YHY analyzed the data and wrote the manuscript. YXY, WD, and HW revised the manuscript. All authors have read and agreed to the published version of the manuscript.

## Conflict of Interest

The authors declare that the research was conducted in the absence of any commercial or financial relationships that could be construed as a potential conflict of interest.

## Publisher’s Note

All claims expressed in this article are solely those of the authors and do not necessarily represent those of their affiliated organizations, or those of the publisher, the editors and the reviewers. Any product that may be evaluated in this article, or claim that may be made by its manufacturer, is not guaranteed or endorsed by the publisher.

## Footnotes

1
http://passport.pepper.snu.ac.kr/?t=PGENOME

2
http://pepperhub.hzau.edu.cn/

3
https://www.arabidopsis.org/

4
https://phytozome-next.jgi.doe.gov/

5
http://pfam.sanger.ac.uk/

6
https://bioedit.software.informer.com/

7
http://web.expasy.org/protparam/

8
https://www.megasoftware.net/

9
https://sourceforge.net/projects/mev-tm4/

10
https://www.originlab.com/

11
https://www.genscript.com
